# Serum Elabela Level Significantly Increased in Patients with Complete Heart Block

**DOI:** 10.21470/1678-9741-2019-0461

**Published:** 2020

**Authors:** Armağan Acele, Atilla Bulut, Yurdaer Donmez, Mevlut Koc

**Affiliations:** 1 Department of Cardiology, University of Health Sciences - Adana Health Practice and Research Center, Adana, Turkey.

**Keywords:** Atrioventricular Block, Pacemaker Artificial, Cardiac Output, C-Reactive Protein, Cholesterol, HDL

## Abstract

**Objective:**

To investigate the change in serum Elabela level, a new apelinergic system peptide, in patients with complete atrioventricular (AV) block and healthy controls.

**Methods:**

The study included 50 patients with planned cardiac pacemaker (PM) implantation due to complete AV block and 50 healthy controls with similar age and gender. Elabela level was measured in addition to routine anamnesis, physical examination, and laboratory tests. Patients were divided into two groups, with and without AV block, and then compared.

**Results:**

In patients with AV block, serum Elabela level was significantly higher and heart rate and cardiac output were significantly lower than in healthy controls. Serum Elabela level was found to be positively correlated with high-sensitive C-reactive protein and N-terminal pro-brain natriuretic peptide (NT-proBNP) levels, but negatively correlated with heart rate, high-density lipoprotein cholesterol, and cardiac output. In linear regression analysis, it was found that these parameters were only closely related to heart rate and NT-proBNP. Serum Elabela level was determined in the patients with AV block independently; an Elabela level > 9.5 ng/ml determined the risk of complete AV-block with 90.2% sensitivity and 88.0% specificity.

**Conclusion:**

In patients with complete AV block, the serum Elabela level increases significantly before the PM implantation procedure. According to the results of our study, it was concluded that serum Elabela level could be used in the early determination of patients with complete AV block.

**Table:** 

Abbreviations, acronyms & symbols	
**AT1**	**= Angiotensin II receptor type 1**
**AV**	**= Atrioventricular**
**HDL**	**= High-density lipoprotein**
**Hs-CRP**	**= High-sensitive C-reactive protein**
**LDL**	**= Low-density lipoprotein**
**LV**	**= Left ventricular**
**NT-proBNP**	**= N-terminal pro-brain natriuretic peptide**
**PM**	**= Pacemaker**
**RAA**	**= Renin-angiotensin-aldosterone**
**ROC**	**= Receiver operating characteristic**

## INTRODUCTION

The cardiovascular system, cardiac myocytes, and cardiac conduction system are under the influence of many systemic and local bioactive peptides and the neural and hormonal systems. The renin-angiotensin-aldosterone (RAA) system, sympathetic nervous system, parasympathetic nervous system, and natriuretic peptide system are well known and previously researched. These systems are interrelated and are often activated for the purpose of protecting the cardiovascular condition, and they can be used in the diagnosis and treatment of cardiovascular diseases. The apelinergic system has been reported to play an important role in the modulation of the cardiovascular system via apelin peptide and APJ receptor^[1,2]^. The APJ receptor has 31% similarity to the final receptor of the RAA system, the angiotensin II receptor type 1 (AT1)^[3]^. Elabela and apelin antagonize the RAA system and, therefore, play a crucial role in the prevention of cardiovascular disease and slowing disease progression^[3]^. In recent studies, apelin and these related peptide receptors have been shown to have cardioprotective effects in atherosclerosis and myocardial infarction, heart failure, and pulmonary arterial hypertension^[4-6]^.

Recently, a peptide called Elabela, which binds to the same receptor as apelin and has similar effects, has been found^[7]^. Elabela's cardiac positive effect is more effective than apelin. The effects of these peptides begin in the embryonic stage and are associated with angiogenesis. In the literature, studies have reported that Elabela is associated with many cardiovascular conditions and diseases^[3]^. Apelinergic system activity is increasing especially in cases of remodeling, heart failure, and arterial hypertension after myocardial infarction and it has positive effects by blocking the RAA system^[3]^. In a study on conscious rats, intravenous, intracoronary, or intraperitoneal administration of apelin or Elabela showed a significant increase in heart rate^[8-11]^. This effect is not related to the AT1 receptor and may be associated with a positive chronotropic effect on the cardiovascular system. However, to the best of our knowledge, in the literature, there is no study investigating the serum Elabela or apelin levels in patients with cardiac conduction system disease or third-degree atrioventricular (AV) block who require cardiac pacemaker implantation. We hypothesized that an increase in the activity of the apelinergic system may occur in response to bradycardia and, therefore, an increase in serum apelin or Elabela levels may be considered.

Hence, in our study, we aimed to investigate whether there is a change in serum Elabela level, a new apelinergic system peptide, in patients with third-degree AV block compared to healthy controls.

## METHODS

### Subjects Identification

This cross-sectional study included 50 patients (24 males, 26 females, mean age 52.7±6.8 years) who were diagnosed with a third-degree AV block and, therefore, underwent conventional pacemaker implantation and 50 healthy individuals (22 males, 28 females, mean age 51.6±6.0 years). The study was conducted in a single center between October 2018 and March 2019 at the Adana City Training and Research Hospital, Turkey. The duration of bradycardia before AV block was unknown. All patients with complete and permanent AV block were included in this study before pacemaker implantation. Pacemaker implantation indication was decided according to the European Society of Cardiology Cardiac Pacing and Cardiac Resynchronization Guidelines^[12]^. After the diagnosis of complete AV block, all patients were evaluated in terms of secondary reasons for a complete algorithm (laboratory tests, medical treatment history, echocardiography, and ischemia evaluation) to determine the causes of reversible complete AV block. Patients who received medications that have an effect on the AV node and patients with acute cardiac ischemia or endocrinological problems were excluded from the study. Patients with estimated glomerular filtration rate < 60 mL/min/1.73m^2^ or > 30 mg/L of proteinuria were excluded. Patients with secondary hypertension, renal artery stenosis, familial hyperlipidemia, moderate or severe valvular heart disease, known heart failure, and nephrectomy were also excluded. This study followed the recommendations of the ethics principles published in the Declaration of Helsinki, developed by the World Medical Association - WMA and approved by the local ethics committee. The clinical information for the informed consent was explained to the patients in detail, and patients were included in the study after consent was obtained. Anamnesis was taken and detailed physical examinations were performed. Heart rate, systolic blood pressure, and diastolic blood pressure were measured. The demographic data were recorded including age, sex, hypertension, and the presence of diabetes mellitus. The weight and height of all cases were measured.

### Ethical Approval

The study was approved by the Çukurova University School of Medicine Local Ethics Committee (approved at October 5^th^ 2018, decision number 81), and the requirement for informed consent was waived.

### Laboratory Parameters

Routine laboratory parameters of all subjects (glucose, high-sensitive troponin I, N-terminal pro-brain natriuretic peptide [NT-proBNP], renal functions, lipid parameters, high-sensitive C-reactive protein [Hs-CRP], and complete blood count) were analyzed. Serum Elabela levels were determined using commercial kits (Sunred Biological Technology, Shanghai, China). Elabela-32 isoform was measured. The kit used a double-antibody sandwich enzyme-linked immunosorbent assay - ELISA to assay the level of Elabela in blood samples. According to the manufacturer, this assay has inter-assay coefficients of variation < 12% and intra-assay coefficients of variation < 10%. All of the abovementioned tests were performed from blood samples that were taken at the 24^th^ hour of hospital admission.

### Echocardiographic Evaluation

Doppler and two-dimensional echocardiographic evaluations were performed using an echocardiography device (EPIQ 7; Philips Healthcare, Andover, Massachusetts, United States of America). The standards of the American Society of Echocardiography were used for all measurements. Biplane Simpson’s method was used for the calculation of left ventricular ejection fraction^[13]^. Cardiac output measurement was calculated with the stroke volume ´ heart rate/1000 formula^[13]^.

### Statistical Analysis

All analyses were performed using the IBM SPSS Statistics (Chicago, Illinois, United States of America) statistical software package, version 22.0. The distribution of continuous variables was evaluated by the Kolmogorov-Smirnov test. Continuous variables in-group data were expressed as mean ± standard deviation. Categorical variables were expressed by number and percentage. Continuous variables that showed normal distribution were compared using the Student’s *t*-test, whereas the Mann-Whitney U test was used to compare differences between two independent groups when the dependent variable was either ordinal or continuous, but not normally distributed. Chi-square (χ2) test was used to compare categorical variables. Pearson and Spearman correlation analysis evaluated the existence of a relationship between countable parameters. In the univariate analysis, statistically significant parameters related to serum Elabela level were included in the multivariate model and multivariate linear regression analysis was performed. A receiver operating characteristic (ROC) curve analysis was performed to re-evaluate the markers that are independent of detecting patients with AV block and to determine the limit value of these markers. The value of the area under the curve was used as a measure of the accuracy of the test. Statistical significance was accepted if *P*<0.05.

## RESULTS

Serum Elabela measurements were successfully obtained from all patients. The study population was divided into two groups as patients with complete AV block and healthy controls. Except for the heart rate, it was found that all parameters were similar between the two groups (Table 1). Heart rate was significantly lower in patients with AV block. According to laboratory data, all laboratory parameters except Elabela and cardiac output were similar between the groups (Table 1). Serum Elabela levels were found to be higher in patients with complete AV block than in healthy controls (Table 1). Cardiac output was significantly lower in patients with AV block than in healthy controls (Table 1).

**Table 1:** Basal characteristics and laboratory parameters of study groups with and without atrioventricular (AV) block.

	Patients with AV block n=50	Healthy controls n=50	*P*-value
Age (years)	52.7±6.8	51.6±6.0	0.391
Gender (male/female)	24/26	22/28	0.181
Heart rate (beats/min)	45.3±3.6	81.2±6.8	<0.001
Systolic blood pressure (mmHg)	122.1±22.7	115.3±14.9	0.077
Diastolic blood pressure (mmHg)	78.6±10.9	75.9±8.8	0.172
Weight (kg)	76.5±8.8	77.3±9.9	0.658
Height (cm)	167±8.1	170±8.8	0.072
Hypertension (n, %)	24 (48%)	18 (36%)	0.117
Diabetes mellitus (n, %)	5 (10%)	0 (0%)	0.117
Smoking (n, %)	24 (48%)	22 (44%)	0.457
Glucose (mg/dl)	91.6±11	91±9.1	0.766
Total cholesterol (mg/dL)	201±37	198±30	0.679
LDL cholesterol (mg/dL)	133±32	130±26	0.630
HDL cholesterol (mg/dL)	42.5±11.9	47.1±12.8	0.062
Triglycerides (mg/dL)	185±81	165±215	0.544
Urea (mg/dL)	32.1±10.5	29.9±5.2	0.198
Creatinine (mg/dL)	0.78±0.16	0.79±0.25	0.700
Hs-CRP (mg/L)	0.36±0.43	0.38±0.24	0.808
NT-proBNP (pg/ml)	113±222	80.9±24.5	0.299
Troponin I (ng/ml)	0.066±0.259	0.058±0.177	0.858
White blood cell (1000/mm^3^)	8.83±7.62	7.17±1.67	0.246
Hemoglobin (g/dL)	13.4±1.33	13.2±0.96	0.436
LV ejection fraction (%)	63.5±4.4	62.7±3.5	0.315
Cardiac output (L/min)	2.80±0.52	4.73±0.63	<0.001
Elabela (ng/ml)	13.8±5.52	2.79±0.85	<0.001

HDL=high-density lipoprotein; Hs-CRP=high-sensitive C-reactive protein; LDL=low-density lipoprotein; LV=left ventricular; NT-proBNP=N-terminal pro-brain natriuretic peptide

Serum Elabela level was found to be positively correlated with Hs-CRP and NT-proBNP levels, but negatively correlated with heart rate, high-density lipoprotein cholesterol, and cardiac output (Table 2). In linear regression analysis, it was found that these parameters were only closely related to heart rate and NT-proBNP (Table 2).

**Table 2:** The parameters associated with serum Elabela levels.

	Univariate analysis		Multivariate analysis	
	*P*-value	r	*P*-value	β
Heart rate (beats/minute)	<0.001	- 0.811	<0.001	- 0.723
HDL cholesterol (mg/dL)	0.042	- 0.203	0.134	- 0.056
Hs-CRP (mg/L)	0.035	0.210	0.256	0.026
NT-proBNP (pg/ml)	<0.001	0.560	<0.001	0.441
Cardiac output (L/min)	<0.001	- 0.682	0.863	- 0.014

HDL=high-density lipoprotein; Hs-CRP=high-sensitive C-reactive protein; NT-proBNP=N-terminal pro-brain natriuretic peptide in multivariate analysis.

**Fig. 1:**
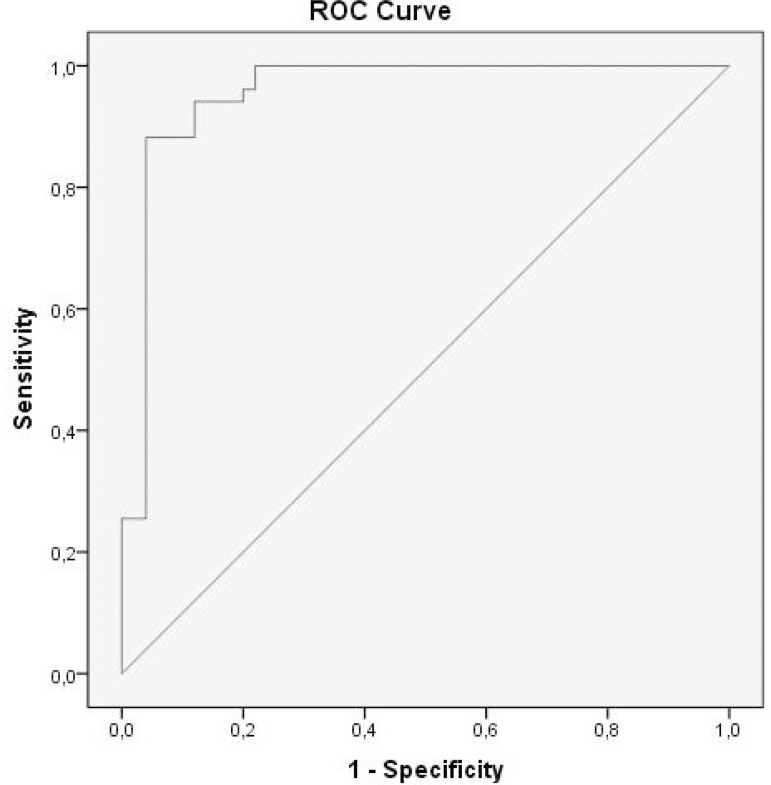
Sensitivity and specificity of Elabela level for the determination of patients with complete atrioventricular block. ROC=receiver operating characteristic

When the ROC analysis was performed for the importance of serum Elabela level in determining the patients with complete AV block, the area under the curve was found to be 0.905. In this analysis, when a limit value for serum Elabela level was taken as 9.5 ng/ml, it determined the patients with complete AV block with 90.2% sensitivity and 88% specificity (Figure 1).

## DISCUSSION

The most important finding of our study was that the serum Elabela value was significantly increased in patients with complete AV block compared to healthy controls. To the best of our knowledge, our study was the first to show that serum Elabela value was increased in patients with complete AV block. In addition, we found that the serum Elabela level above 9.5 ng/mL determined the presence of complete AV block with acceptable sensitivity and specificity. There was a close relationship between serum Elabela level and heart rate.

Studies on the effects of apelin and Elabela on the cardiovascular system showed that they: i) contribute to the formation of heart and angiogenesis in the embryogenic period; ii) have inotropic effects; iii) cause vasodilatation in both systemic and pulmonary vascular systems; iv) cause reduction or deceleration in diseases, leading to cardiac hypertrophy and fibrosis; v) reduce peripheral vascular disease; and vi) improve heart failure and myocardial infarction clinic^[3]^. Therefore, it is thought that this may be a treatment method because of all these positive and cardiovascular protective effects^[14]^. Serum Elabela or apelin levels have been studied in patients with atherosclerosis and myocardial infarction, heart failure, and pulmonary arterial hypertension^[4-6]^. To the best of our knowledge, there is no study on serum apelin or Elabela level in AV block patients. These peptides, which are usually cardiac-protective and which are synthesized for the purpose of secondary protection against various cardiovascular diseases may also have a protective effect on patients with AV block.

Bradycardia and associated cardiac output reduction in patients with AV block cause a reduction of perfusion in organs such as the heart, brain, kidney, and liver. In order to compensate for this pathophysiological condition and to ensure organ perfusion, the activity of RAA and the sympathetic nervous system is increased, and the blood pressure of these patients is improved. The reflex response of the apelinergic system to the formation of the AV block and bradycardia is not clear. Several experimental studies have reported an increase in heart rate with apelin and Elabela^[8-11]^. However, in a previous study, there is also information about the absence of an increased heart rate^[15]^. As a result of these studies, apelin receptors may be present in cardiac pacemaker cells and the increase in heart rate may be different from other systems. As a result of these studies, many studies are needed to demonstrate the net effects of apelin, Elabela, and other APJ receptor stimulants on cardiovascular system regulation and to demonstrate their pathophysiological effects. Although our study did not give information about Elabela and its cardiac pathophysiological effects, it was shown that serum Elabela level was significantly increased in patients with AV block compared to healthy controls. This is the first study in the literature on this subject. Increased Elabela level in patients with AV block was thought to be a reflex condition with the aim of increasing cardiac output. Elabela serum level may be increased to augment cardiac output and heart rate in response to bradycardia. Although this hypothesis is ambitious, we have not shown it at a pathophysiological and cellular level. It would be interesting to see serum Elabela levels in patients with sinus bradycardia or bradycardic atrial fibrillation in future studies. It is a big question that serum Elabela is increased just for the bradycardia or concomitantly reduced output. Is it possible to detect low levels of serum Elebela in patients with tachycardia with the same logic? We are further interested in these issues. Our findings can be evaluated with new studies similar to ours and with different and more populations.

This study has some limitations. Although the results of our study were significant, the number of patients was insufficient. Although biochemical measurement was performed in our study, we did not study the APJ receptor level of tissue samples. The examination of similar findings in the cardiac conduction system or myocytes could be more meaningful. There is a close relationship between the apelinergic system and RAA; in our study, no biochemical analysis was performed on the RAA system. A more enlightening result could be obtained if a biochemical evaluation of the RAA system was performed. In our study, Elabela levels were only measured before pacemaker implantation. Elabela may be elevated due to bradycardia for cardiac protection, so a more revealing result could be obtained if the serum level was measured after pacemaker implantation. New studies should be performed in which the RAA system is examined, tissue samples are examined, and the Elabela level is evaluated after pacemaker implantation.

## CONCLUSION

Serum Elabela value increases compared to healthy controls in patients with complete AV block. This may be a finding of up-regulation in the apelinergic system to have positive effects on the cardiovascular system in patients with complete AV block. High serum Elabela levels may also be the preliminary finding of a future AV block in patients without AV block or intermittent AV block. For this purpose, the serum Elabela level can be used with a limit value of 9.5 ng/mL. This can also be used as a follow-up parameter in other similar diseases. Patients with elevated serum Elabela levels may be closely examined frequently for a new developed AV block. However, this finding should be confirmed by further studies, where more patients are included.

**Table:** 

Authors' roles & responsibilities	
AA	Substantial contributions to the conception or design of the work; or the acquisition, analysis, or interpretation of data for the work; drafting the work or revising it critically for important intellectual content
AB	Substantial contributions to the conception or design of the work; or the acquisition, analysis, or interpretation of data for the work; drafting the work or revising it critically for important intellectual content
YD	Substantial contributions to the conception or design of the work; or the acquisition, analysis, or interpretation of data for the work; drafting the work or revising it critically for important intellectual content
MK	Drafting the work or revising it critically for important intellectual content; agreement to be accountable for all aspects of the work in ensuring that questions related to the accuracy or integrity of any part of the work are appropriately investigated and resolved; final approval of the version to be published
